# Detailed Characterization of the Cell Wall Structure and Composition of Nordic Green Microalgae

**DOI:** 10.1021/acs.jafc.2c02783

**Published:** 2022-07-27

**Authors:** Olivia Spain, Christiane Funk

**Affiliations:** Department of Chemistry, Umeå University, 90187 Umeå, Sweden

**Keywords:** microalgae, cell wall, FTIR, cryo-XPS, imaging

## Abstract

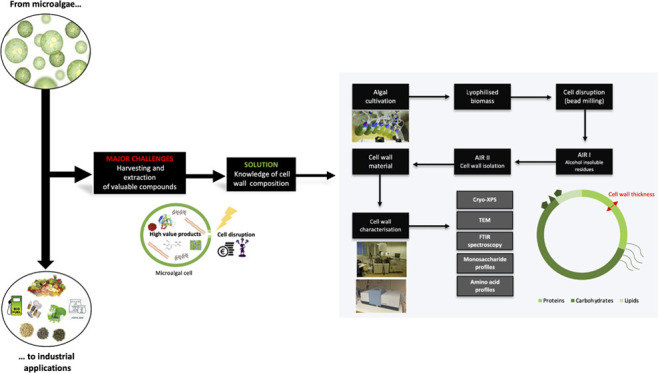

Green microalgae are attractive to food, pharmaceutical, and biofuel industries due to the promising and diverse properties of their intracellular components. In current biotechnological applications, however, clear bottlenecks are the cell disruption and cell harvesting steps. Challenges in both of these processes are directly linked to the properties of the microalgal cell wall. The aim of this study was to explore the cell wall compositions and morphologies of four Nordic microalgal strains (*Chlorella vulgaris* (13-1), *Scenedesmus* sp. (B2-2), *Haematococcus pluvialis*, and *Coelastrella* sp. (3-4)) and their changes in relation to logarithmic and stationary growth phases. Transmission electron microscopy imaging enabled us to visualize the cell walls and to observe structural elements such as spines, microfibrillar hairs, or layers. Using cryogenic X-ray photoelectron spectroscopy, we quantified lipid, protein, and polysaccharide content of the outer surface of the microalgal cell wall in cultures. Fourier transform infrared spectroscopy highlighted changes between growth phases within the polysaccharide and protein fractions of the cell wall. Very prominent differences were observed in sugar and protein composition of the *Scenedesmus* sp. (B2-2) cell wall compared to the cell walls of the other three Nordic strains using trimethylsilyl derivatization.

## Introduction

Microalgae are a very promising source of valuable bioactive compounds such as polymers, vitamins, peptides, carotenoids, and sterols.^1^ These compounds are well studied and can be used in a wide variety of applications including in food, pharmaceutical, cosmetic, bioenergy, and biofertilizer industries. Certain microalgal compounds can act as antimicrobials, antioxidants, and anticancer agents or can have anti-inflammatory, antiobesity, and even antidiabetic activity.^2^ Although these impressive properties of microalgae are well known, their utilization on larger scales remains difficult as we are yet to find efficient downstream treatments that allow for high extraction yields. In current algal treatment processes, there are indeed two main bottlenecks: the harvesting of the algae and the extraction of the high-value compounds from within the cells. These two steps are costly in energy and time and are greatly dependent on the cell wall of the algae.^3^ Microalgae are usually surrounded by a remarkably thick and resistant cell wall, whose structure and molecular components display great diversity depending on the strain. While the cell wall properly fulfills its protective role and ensures cell viability, it also limits the bioaccessibility and therefore the extractability of the compounds enclosed within the cells.^3^ Despite its apparent importance in biotechnological applications, currently, very little is known about the composition and ultrastructure of the cell wall of the microalgae used in biotechnological applications. Furthermore, the cell wall parameters vary between genus, species, and even within the same strain depending on the growth conditions and life stage of the cell,^4^ so cell wall studies have to be conducted on each industrially relevant strain and at different growth stages. In 2017, Baudelet et al. wrote a very detailed state of the art, providing insight on current knowledge of cell wall structure and composition.^4^ We summarized this information in our more recent review.^5^

In this study, we focus on four strains of our Nordic microalgae culture collection,^6^ namely, *Chlorella vulgaris* (strain ID 13-1), *Scenedesmus* sp. (strain ID B2-2), *Haematococcus pluvialis*,^7^ and *Coelastrella* sp. (strain ID 3-4), all isolated around Umeå, Northern Sweden. These strains represent microalgal genera commonly used in biotechnological applications; our data are therefore highly valuable for a broad readership. However, compared to strains, who conventionally are used in warm and sunny climates, these Nordic strains live and thrive under remarkably harsh growth conditions (short photoperiod and very low temperatures in the winter, long photoperiod at relatively low temperatures in the summer). Their impressive resistance to these conditions could be due to a thicker, more rigid cell wall that acts as a barrier against environmental factors. The strains used in this study were isolated in 2017 and have since then proven to be of industrial interest for their capacity to produce high quantities of biomass, treat wastewaters, and even metabolize the contaminants^8^ and produce large quantities of lipids. To be able to fully exploit the potential of Nordic microalgae, we need to understand their specific and unique cell wall properties.

The genus *Chlorella* is the most common and best-studied group of green microalgae for biotechnological applications and is characterized by small, solitary, spherical, and nonmotile cells. Of our various Nordic *Chlorella* species, *C. vulgaris* (strain ID 13-1) is by far the best performing in biomass generation; it is resistant to cold stress,^9^ grows hetero- and mixotrophically,^10^ and efficiently takes up nutrients and pharmaceuticals from wastewater and is even able to metabolize these contaminants.^8^

*Coelastrella* sp. (strain ID 3-4) was isolated and identified for the first time in Sweden in 2016.^6^ The fast-growing strain is characterized by its large spherical cells of 5–15 μm in diameter and its capacity to flocculate and to produce high quantities of lipids (on average, 30.8% of the dry weight of the biomass corresponds to lipids^6^).

The genus *Scenedesmus* is commonly used in algal farms, and our findings will therefore be of importance for various applications. It is composed of colonial, green microalgae species, which form 2 to 16 cell colonies. The Nordic strain *Scenedesmus* sp. (strain ID B2-2) is a very robust and cold-tolerant strain that forms colonies of four cells and produces high amounts of biomass and lipids (>30% of the biomass).^9^

*H. pluvialis* is an excellent natural source of astaxanthin, a carotenoid pigment. Its antioxidant properties make it a high-value product for use in food, cosmetics, and pharmaceuticals. Astaxanthin accumulates in *Haematococcus* cells when they are exposed to stress, during which they transform from green vegetative cells to red cysts. Extracting astaxanthin from these cysts is currently very challenging because of the thick and structurally sophisticated cell wall.^7^

Generally, in current microalgal technologies, it is considered advantageous to harvest the algae in the stationary phase, as not only do older cells usually contain higher quantities of lipids, protein, and vitamins, but they also have lower mobility and lower ζ-potential.^11^ However, these parameters are different depending on the microalgae in question, and some strains may present more interesting/exploitable properties in the logarithmic growth phase as opposed to the stationary growth phase. To learn more about the microalgal cell wall, we chose to study its composition in optimal growth conditions as well as in the stationary phase, to get an overall understanding of how the cell wall evolves over time. This will give us the potential to identify a time in the growth where there is a good balance between breakable cell wall properties and valuable compound content for all of our strains.

## Materials and Methods

### Cell Culture

The four microalgal strains *C. vulgaris* (13-1), *Coelastrella* sp. (3-4), *Scendesmus* sp. (B2-2), and *H. pluvialis* ^6,7^ were preinoculated in 100 mL Erlenmeyer flasks filled up to 30% volume with BG11 medium.^12^ The flasks were placed in a closed orbital shaker at 115 rpm, 25 °C, and 100 μmol/m^2^/s of white light. After 7 days, the whole preinoculum was transferred into 1 L bottles, filled with 900 mL of BG11 medium, and bubbled with a mixture of air and CO_2_. The starting OD at λ = 750 nm was approximately 0.1, and the cultures were left to grow for 10 days, with a first sample taken on day 5. Each culture was observed under the light microscope (Leica DMi1, 40× magnification) to exclude bacterial contamination. The growth curves indicate the algae cultures to be in an exponential growth phase from day 2 of cultivation, and that they enter a stationary phase after 9 days of growth. On growth days 5 and 10, the biomass was harvested by centrifugation at 15 °C and 20,000*g* for 20 min. The biomass was freeze-dried, and the resulting material was used for the following steps.

### Cell Wall Isolation (AIR I)

To isolate the cell wall, 80% ethanol was added to the dry algal material in a ratio of 500 μL of ethanol per 10 mg of algal biomass. The samples were heated for 30 min at 95 °C and cooled down on ice before centrifugation at 21,000*g* for 10 min. The pellet was resuspended in 70% ethanol, vortexed, and heated for another 30 min at 95 °C. This step was repeated twice. After centrifugation, 80% methanol was added to the pellet to extract phenolics. The mixture was vortexed, centrifuged, and the pellet was resuspended in chloroform/methanol in a 1:1 ratio, vortexed, and left to incubate at room temperature for 15 min. After centrifugation, the pellet was washed three times with 100% acetone, centrifuged for 10 min at 21,000*g*, and placed in a desiccator overnight. The resulting dry material is referred to as AIR I material.

### Amylase Treatment of Cell Wall Material (AIR II)

The AIR I material was resuspended in 0.1 M potassium phosphate buffer, in a ratio of 10 mg of cell wall material per mL of buffer.^13^ Per gram of cell wall material, 1 μL of 0.01% sodium azide, 1000 units (100 μL) of α-amylase, and 8 μL of amyloglucosidase were added to each tube. The tubes were placed in a shaker for digestion at 37 °C for 24 h. The shaker rotated horizontally as to avoid the accumulation of cell wall material in the lid of the tube. On the next day, the tubes were centrifuged at 18,000*g* for 10 min. The resulting supernatant was stored at −20 °C for glucose analysis. The pelleted cell wall material was resuspended in 0.1 M potassium phosphate buffer, to which per mL of buffer 1 μL of 0.01% sodium azide had been added, and per gram of cell wall material 100 μL of α-amylase and 8 μL of amyloglucosidase have been added. The digestion was continued for an additional 24 h in the horizontal shaker. This process was repeated three times, and each supernatant was stored at −20 °C for glucose analysis. The pellet was then washed three times, once with buffer, once with water, and once with acetone, and then placed in a desiccator overnight.

### Cryogenic X-ray Photoelectron Spectroscopy

To perform cryogenic X-ray photoelectron spectroscopy (cryo-XPS), the preinoculum of the four strains after 7 days was transferred into 1 L Erlenmeyer flasks filled with 600 mL of BG11 medium. After 5 or 10 days, the cells were pelleted and washed with phosphate buffer, and the fresh biomass was used for the experiment according to Gojkovic et al.^14^ XPS spectra were recorded with a Kratos Axis Ultra DLD electron spectrometer. A monochromated Al Kα source operated at 150 W, a hybrid lens system with a magnetic lens providing an analysis area of 0.3 mm by 0.7 mm, and a charge neutralizer were used for the measurements. The binding energy (BE) scale was referenced to the C 1s line of aliphatic carbon, set at 285.0 eV. Casa XPS and Vision2 Kratos software were used for the processing of the obtained spectra.

### Fourier Transform Infrared (FTIR) Spectroscopy Characterization

Fourier transform infrared (FTIR) spectroscopy was performed on dry cell wall material, according to the protocol by Gorzsás and Sundberg.^15^ Briefly, ca. 10 mg of dry sample material was mixed with ca. 390 mg of KBr and analyzed by diffuse reflectance FTIR spectroscopy (DRIFTS), under vacuum conditions (4 mbar), using a Bruker IFS 66v/S instrument (Bruker Optik GmbH, Ettlingen, Germany). Spectra were recorded over the range of 400–4000 cm^–1^ at a spectral resolution of 4 cm^–1^, with 128 scans co-added. The background was pure KBr. Spectra were exported as ASCII files and imported into MATLAB to be processed by the free open-source MATLAB-based script provided by the Vibrational Spectroscopy Core Facility at Umeå University, Sweden (https://www.umu.se/en/research/infrastructure/visp/downloads/). The spectra were baseline-corrected using asymmetric least-squares fitting with λ = 10.000.000, and *p* = 0.001. Spectra were normalized to the amide I band (using the Region MinMax option for the 770–1970 cm^–1^ spectral range) and slightly smoothed using Savitzky–Golay smoothing (with a first-order polynomial and a frame of 5).

### Monosugar Analysis by Trimethylsilyl Derivatization (TMS)

Cell wall material (500 μg) was weighed in screw-capped tubes. A standard range was used with 10, 20, 50, and 100 μg of TMS standard solution. Inositol (30 μg) was added to every tube as an internal standard. The tubes were placed in a heating block under a stream of nitrogen at 60 °C and left to dry for 30 min. Sulfuric acid (72%, 35 μL) was added to each tube, and they were placed in a sonicator for 30 min. The tubes were incubated for 1 h at room temperature. Water (980 μL) was then added to each tube, and all tubes were placed in a heating block at 80 °C for 2.5 h. The tubes were left to cool and were centrifuged at 21,000*g* for 5 min. Supernatant (1 mL) was collected and neutralized with CaCO_3_. The samples were centrifuged at 21,000*g* for 10 min, and 800 μL of the supernatant was transferred into an Eppendorf tube. This step was repeated twice, and the final supernatant was transferred into glass tubes. The tubes were dried in a heating block at 60 °C and under a stream of nitrogen for 30–45 min. They were then placed in a desiccator with a phosphorous pentoxide desiccant and left to dry at room temperature overnight. The following day, 600 μL of 2 M HCl/MeOH was added to each tube. The tubes were then immediately flushed with a stream of nitrogen, closed with Teflon coated caps, and incubated for 24 h at 85 °C. After 24 h, the samples were cooled at room temperature and then placed under a stream of nitrogen for 10 min at 40 °C to let the solvent evaporate. Next, 300 μL of methanol was added and evaporated under a stream of nitrogen for 10 min. This step was repeated three times so that the samples were completely dry after the third evaporation.

In the final step (silyation), 200 μL of Tri-sil reagent (HMDS+TMCS+ Pyridine 2:1:10 (v/v/v)) was added to each tube. The tubes were closed and heated for 20 min at 80 °C. After cooling, the solvent was evaporated under a stream of nitrogen. Hexane (1 mL) was added to each tube, and the content was then vortexed and transferred to an Eppendorf tube. The tubes were centrifuged for 5 min at 20,000*g*, and the content was filtered through glass wool. The solvent (200 μL) was transferred to a GC micro vial. The samples were analyzed by gas chromatography–mass spectrometry (GC–MS) (7890A/5975C; Agilent Technologies) according to Sweeley et al.^16^ The column and oven program used for this experiment are described by Latha Gandla et al.^17^

### Amino Acid Analysis, Standards, and Calibration Curve

Amino acid standards (alanine, arginine, aspartic acid, cysteine, glutamic acid, glycine, histidine, isoleucine, leucine, lysine, methionine, phenylalanine, proline, serine, threonine, tyrosine, valine, glutamine, asparagine, GABA, citrulline, ornithine, taurine, tryptophan, 5-HTP, norvaline, and kynurenine) were purchased from Sigma (St. Louis, MO). Isotopically labeled amino acid standards (alanine (^13^C_3_,^15^N), arginine (^13^C_6_, ^15^N_4_), aspartic acid (^13^C_4_, ^15^N), cystine (^13^C_6_, ^15^N_2_), glutamic acid (^13^C_5_, ^15^N), glycine (^13^C_2_, ^15^N), histidine (^13^C_6_, ^15^N_3_), isoleucine (^13^C_6_, ^15^N), leucine (^13^C_6_, ^15^N), lysine (^13^C_6_, ^15^N_2_), methionine (^13^C_5_, ^15^N), phenylalanine (^13^C_9_, ^15^N), proline (^13^C_5_, ^15^N), serine (^13^C_3_, ^15^N), threonine (^13^C_4_, ^15^N), tyrosine (^13^C_9_, ^15^N), valine (^13^C_5_, ^15^N), citrulline (d4), GABA (^13^C_4_), glutamine (^13^C_5_), asparagine(^13^C_4_), ornithine (d6), tryptophan (d8), kynurenine (d4)) were obtained from Cambridge Isotope Laboratories (Andover, MA). Stock solutions of each compound were prepared at a concentration of 500 ng/μL and stored at −80 °C. A 10-point calibration curve (0.01–100 pmol/μL) was prepared by serial dilutions and spiked with internal standards at a final concentration of 5 pmol/μL. Mass spectrometry-grade formic acid was purchased from Sigma-Aldrich (St Louis, MO), and HPLC-grade acetonitrile was purchased from Fisher Scientific (Fair Lawn, NJ).

### Extraction and Hydrolysis of Amino Acids

Cell wall material (5 mg) was used for the analysis of bound amino acids (BAAs). For the hydrolysis of proteins, 1 mL of 6 M HCl was added to each sample. The samples were incubated at 110 °C for 16 h. After incubation, the samples were centrifuged (4 °C, 21,000*g*, for 10 min) and the supernatant containing BAAs was collected. The BAA extract (10 μL) was diluted with 90 μL of Milli-Q water. The diluted sample (20 μL) was transferred to an LC-MS vial and evaporated in a speed vacuum concentrator.

### Amino Acid Derivatization with AccQ-Tag

Extracted samples were derivatized by AccQ-Tag (Waters, Milford, MA) according to the manufacturer’s instructions with the following adjustments: The dried BAA samples were dissolved in 80 μL of AccQ•Tag Ultra Borate buffer spiked with all isotopically labeled internal standards at a final concentration of 0.625 pmol/μL. Finally, 20 μL of the freshly prepared AccQ·Tag derivatization solution was added and the sample was immediately vortexed for 30 s. Samples were kept at room temperature for 30 min followed by 10 min at 55 °C. For each batch, quality control samples and procedure blanks were included. Calibration curves were prepared in the same way as for the samples.

### Amino Acid Quantification by LC-ESI-MSMS

The derivatized samples were analyzed using a 1290 Infinitely system from Agilent Technologies (Waldbronn, Germany), consisting of a G4220A binary pump, a G1316C thermostated column compartment, and a G4226A autosampler with G1330B autosampler thermostat coupled to an Agilent 6460 triple quadrupole mass spectrometer equipped with a jet-stream electrospray source operating in positive-ion mode.

Separation was achieved by injecting 1 μL of each sample onto a BEH C_18_ 2.1 × 100 mm, 1.7 μm column (Waters, Milford, MA) held at 50 °C in a column oven. The gradient eluents used were H_2_O, 0.1% formic acid (A) and acetonitrile, 0.1% formic acid (B) with a flow rate of 500 μL/min. The initial conditions consisted of 0% B, and the following gradient was used with linear increments: 0.54–3.50 min (0.1–9.1% B), 3.50–7.0 (9.1–17.0% B), 7.0–8.0 (17.0–19.70% B), 8.0–8.5 (19.7% B), 8.5–9.0 (19.7–21.2% B), 9.0–10.0 (21.2–59.6% B), 10.0–11.0 (59.6–95.0% B), 11.0–11.5 (95.0% B), 11.5–15.0 (0% B). From 13.0 to 14.8 min, the flow rate was set at 800 μL/min for a faster equilibration of the column.

The MS parameters were optimized for each compound. MRM transitions for the derivatized amino acids were optimized using MassHunter MS Optimizer software (Agilent Technologies, Inc., Santa Clara, CA). The fragmentor voltage was set at 380 V, the cell accelerator voltage at 7 V, and the collision energies from 14 to 45 V. Nitrogen was used as the collision gas. The jet-stream gas temperature was set to 290 °C, with a gas flow of 11 L/min, a sheath gas temperature of 325 °C, and a sheath gas flow of 12 L/min. The nebulizer pressure was set at 20 psi, and the capillary voltage was set at 4 kV. The QqQ was run in Dynamic MRM Mode with 2 min retention time windows and 500 ms cycle scans.

The data were quantified using MassHunter Quantitation software B08.00 (Agilent Technologies, Inc., Santa Clara, CA), and the amount of each amino acid was calculated based on the calibration curves.

### Cell Wall Imaging via Transmission Electron Microscopy (TEM)

For transmission electron microscopy (TEM), the algae were prepared as described in Baker et al.,^18^ with modifications described by Boussardon. Algal cells were fixed in 2.5% glutaraldehyde for 2 h. After fixation, the cells were washed three times with suspending media and postfixed for 2 h in 1% OsO_4_ in water. Dehydration steps were performed through a graded series of ethanol 30, 50, 70, 85, 95, 100% × 2. Thin sections (70 nm) were cut with a Leica EM FC7 ultramicrotome (Leica Microsystems, Inc., Germany) and embedded in Spurr resin (TAAB Laboratories, U.K.). The sections were stained with 5% aqueous uranyl acetate for 45 min followed by lead citrate for 6 min, before being examined under the electron microscope (Talos L120C, Thermo Fisher Scientific).

## Results and Discussion

To analyze the cell wall composition of Nordic algal species and its changes in relation to the growth phase, four different strains important for biotechnological applications were grown under normal conditions. Their growth curves, based on the optical density of each strain, are shown in Supporting Information, Figure S1. *C. vulgaris* (13-1) and *Scenedesmus sp.* (B2-2) reached the stationary phase on day 10, whereas *H. pluvialis* (HP) and *Coelastrella sp.* (3-4) entered the stationary phase on day 9. The highest cell concentration was observed on day 15 for HP (21.48 ×10^6^ cells/mL; OD_680_ = 1.538), followed by *Coelastrella* sp. (3-4) (15.34 × 10^6^ cells/mL; OD_680_ = 1.522), *C. vulgaris* (13-1) (12.64 × 10^6^ cells/mL; OD_680_ = 1.573), and *Scenedesmus* sp. (B2-2) (5.123 × 10^6^ cells/mL; OD_680_ = 1.025), respectively. The highest doubling time was observed for HP (29 h), while the lowest was observed for *Scenedesmus* sp. (B2-2) (43 h). The average doubling time of each strain was calculated during the exponential growth phase. *Coelastrella* sp. had an average doubling time of 34 h and that of *C. vulgaris* (13-1) was 32 h. For this study, we chose to study and compare the cell wall of the microalgae in both the logarithmic and the stationary growth phase. Therefore, for every strain and experiment, the biomass was harvested at growth day 5 and growth day 10.

### Cell Wall Morphology Greatly Varies between Growth Conditions and Strains

The thickness of the microalgal cell wall is a variable that determines cell wall strength^19^ and therefore has a significant influence on cell disruption.^20^ In general, the thickness of the cell wall can vary with growth stages and conditions.

**Figure 1:**
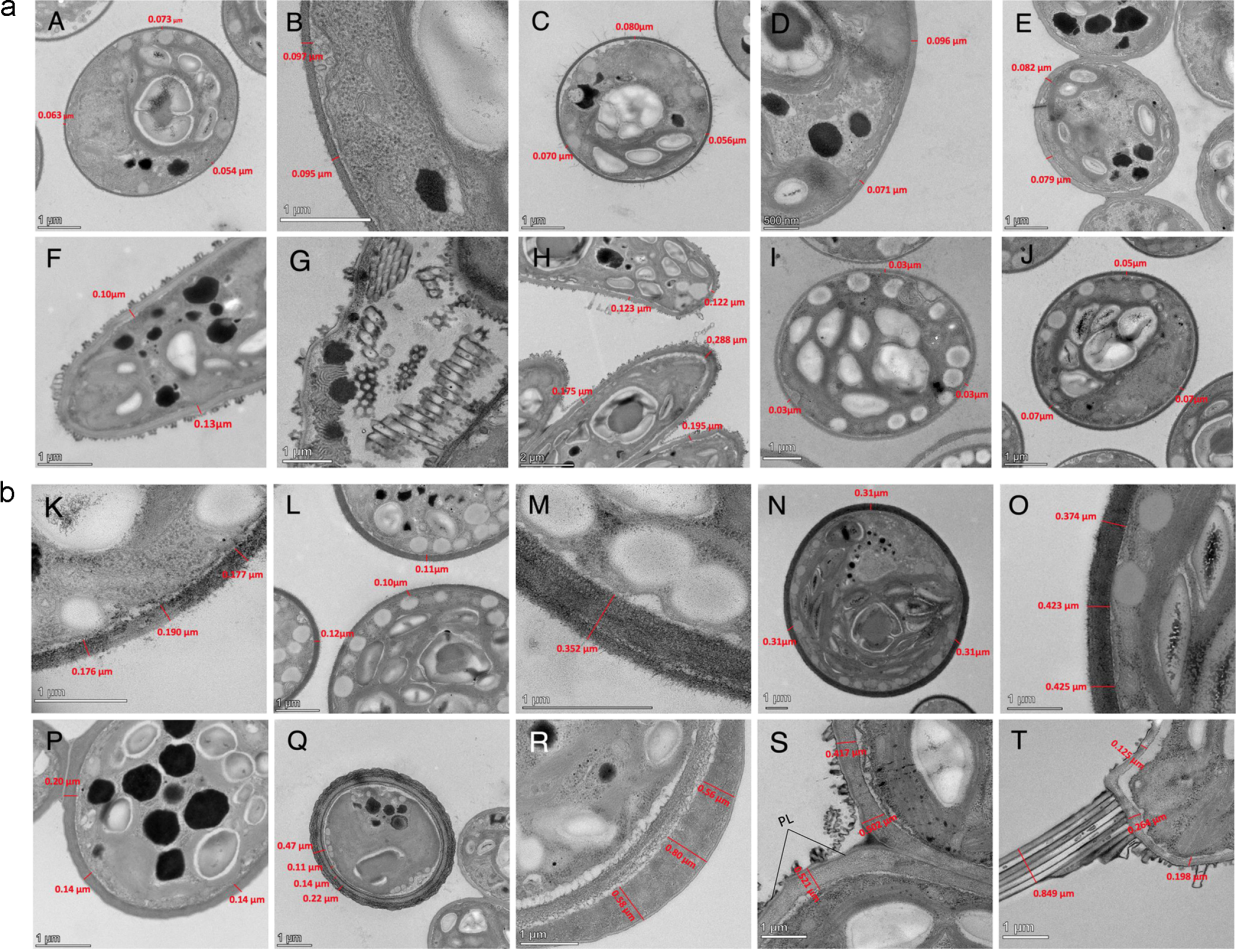
(a) TEM images of the cell walls of *C. vulgaris* (13-1) (A–C), *H. pluvialis* (HP) (D, E), *Scenedesmus sp.* (B2-2) (F–H), and *Coelastrella sp*. (3-4) (I, J) on growth day 5. (b) TEM images of the cell walls on growth day 10 (*C. vulgaris* (13-1)) (K, L, M); *Coelastrella sp*. (3-4) (N, O); *H. pluvialis* (HP) (P, Q, R); *Scenedesmus* sp. (B2-2) (S,T).

The cell wall morphology of the four strains, harvested in both exponential and stationary growth phases, was observed by transmission electron microscopy (TEM) (Figure 1a,b). TEM images revealed clear differences in the cell wall morphology and thickness, between strains and between growth phases. It was measured for each strain on 15 cells in three different places, and the mean is presented in Table 1. The thickness of the cell wall increased in all strains between the exponential growth phase and the stationary growth phase. While it doubled for *Scenedesmus* sp. (B2-2) and *C. vulgaris* (13-1), it tripled for *H. pluvialis*. The most impressive difference in thickness was observed in *Coelastrella* sp. (3-4), whose cell wall was on average 7 times thicker in the stationary phase than in the exponential phase.

**Table 1:** Mean Cell Wall Thickness (μm) at Different Growth Stages

	mean cell wall thickness (μm)	
microalgae	exponential—day 5	stationary—day 10
*C. vulgaris* (13-1)	0.05	0.11
*Coelastrella sp.* (3-4)	0.04	0.31
*H. pluvialis* (HP)	0.08	0.25
*Scenedesmus sp.* (B2-2)	0.11	0.20

When microalgal cells progress into the stationary growth phase and become more exposed to stress, they seem to compensate by producing thicker cell walls. In the exponential growth phase, nonstressed cells of *Chlamydomonas reinhardtii* possess cell walls with one unique layer, whereas when exposed to prolonged stress, the majority of the algal cell walls were composed of two to three layers, and a high percentage of the population had more than three layers.^21^ Although a thicker cell wall is usually expected to be more difficult to break during downstream processing, higher lipid extractability was observed in the older algal cultures, even though the cell walls were composed of more layers than those of the younger cultures.^3,21^ The higher lipid extractability of the older algal cultures suggests a higher vulnerability of the cell wall in the stationary phase. While this was the case for *C. reinhardtii*, the opposite was true for the microalga *Tetraselmis* sp. DS3, whose thinner cell wall was much more easily disrupted by high-pressure gases than the thick-walled thermotolerant microalga *Desmodesmus* sp. F2.^20^

It has previously been reported that the cell wall of *H. pluvialis* goes through five developmental states between the flagellate and the aplanospore state.^22^ During this transformation, the algal cells develop a primary cell wall, a secondary cell wall mostly composed of mannan,^22^ a tripartite crystalline layer, a trilaminar sheath containing algaenan, and different layers of the extracellular matrix. Knowledge of the components of these layers will allow us to target those compounds and break down the cell wall, therefore facilitating the extraction process. On average, the cell wall of Nordic *H. pluvialis* tripled in thickness between growth phases. On day 5, its cell wall was smooth and composed of one unique layer or primary cell wall. In the stationary growth phase, many of the cell walls on the grid displayed multiple layers (either 2 clear layers shown in image S, or up to 5 layers in the aplanospore form, shown in image R).

### Cell Wall Morphology Varies Greatly between Strains

Cell walls can be categorized according to the layers that comprise the cell wall; cell walls can either be made up of one single microfibrillar layer or of an outer and an inner layer. The outer layer can be further composed of one mono-electron-dense layer or three sublayers.^5^ In appearance, the cell wall of the Nordic *Coelastrella* sp. (3-4) strain was smooth and did not contain apparent layers (Figure 1I,J,N,O). However, the cell wall of this strain became very thick and dense in the stationary growth phase.

The surface of the Nordic strain *Scenedesmus* sp. (B2-2) was remarkably different from the other strains of this study, as it was the only one of the four to have structures on the outer surface of the cell (Figure 1F,G,H,S,T). *Scenedesmus* species belong to the Chlorococcales order. Members of this order can exist as single cells; they can also frequently form coenobia of 4 to 16 cells in the later stages of their life cycle. To be able to bind together within the coenobium, the cell walls develop a pectic layer, which delimits each individual cell.^23^ This layer is clearly visible in the Nordic strain (Figure 1b-S, marked PL), and its thickness depended on the culture conditions. The outer surface of the pectic layer was scattered with ornamentations including spines, teeth, and bristles. The marginal cells of the coenobium had long spines at the poles of the cell (Figure 1b-T). These spines are thought to act as a defense mechanism against zooplankton grazing.^24^

The cell wall of the three other strains was smooth. On day 5, the cell wall of HP was composed of one unique layer (Figure 1a-D,E) or primary cell wall. In the stationary growth phase, the cells contained cell walls of multiple layers, either two clear layers (Figure 1b-R) or up to five layers in the aplanospore form (Figure 1b-Q).

While most of the *C. vulgaris* (13-1) cells have a smooth surface, some cells displayed hair-like structures on their surface. Our measurements revealed that these microfibrils have an average thickness of 5 nm. A previous study reported that these hair-like fibers in *Chlorella* species are composed of hyaluronan (linear polysaccharides of β-1,4-glucuronic acid and β-1,3-*N*-acetylglucosamine groups) and may serve as a defense mechanism against the*Paramecium bursaria* Chlorella virus 1.^25^

### FTIR Spectroscopy Points to Changes in Cell Wall Polysaccharides between Growth Phases

**Figure 2:**
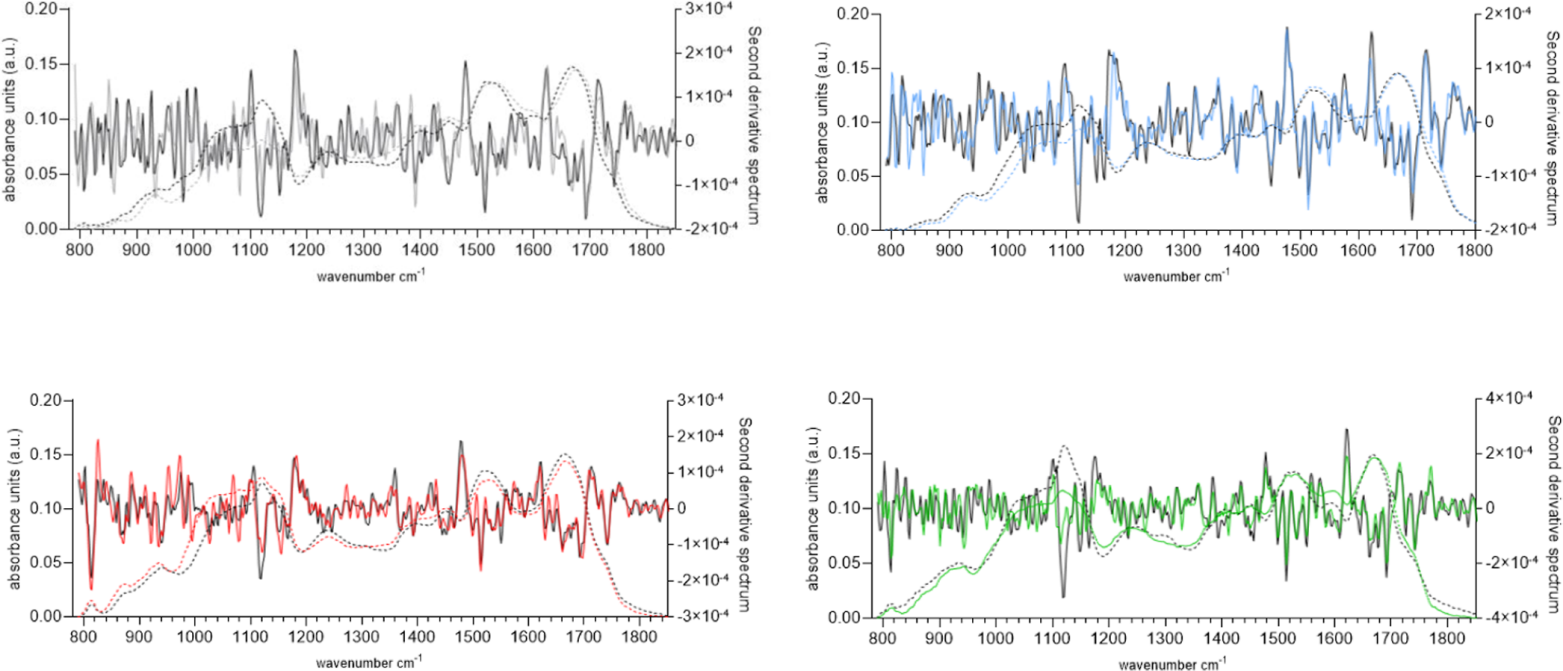
FTIR analysis of *C. vulgaris* (13-1) (A, gray), *Coelastrella* sp. (3-4) (B, blue), *Scenedesmus* sp. (B2-2) (C, red), and *H. pluvialis* (HP) (D, green) on day 5 and day 10 of growth. FTIR spectra (dotted lines) and their second derivatives (full lines) were performed on isolated cell wall material of the four algal strains grown in the logarithmic (colored lines) or stationary (black lines) growth phases.

FTIR spectroscopy has the advantage of being a nondestructive and rapid characterization method that was traditionally used on purified compounds.^26^ More recently, FTIR spectroscopy has also been used to characterize more complex biological systems such as bacteria,^27^ microalgae,^28^ and even higher plants,^29^ and the resulting spectra contain both qualitative and semiquantitative (variability between populations or strains) information.^30^ To characterize the microalgal cell walls and to identify changes between growth phases, FTIR spectroscopy experiments were performed on isolated cell wall material of the four algal strains grown in the logarithmic (represented in colored lines in Figure 2) or stationary (represented in black lines in Figure 2) growth phases. The general shape of the collected spectra revealed the typical carbohydrate (960–1180 cm^–1^) and protein (amide II; 1475–1620 cm^–1^ and amide I; 1620–1710 cm^–1^) regions. The lipid fraction (around 1740 cm^–1^) showed a very weak absorption band, indicating the cell walls only contained very few fatty acids. For comparison between strains, the FTIR spectra (dotted lines in Figure 2) were normalized to the amide I band (i.e to protein content). Additionally, the second derivatives of the FTIR spectra were calculated to highlight the most prominent changes between growth phases (full lines in Figure 2), enhancing the separation of overlapping peaks.^31^

The majority of the variation between growth phases, for the same strain, was seen in the carbohydrate fraction. For *C. vulgaris* (13-1) (Figure 2A), the most prominent changes between growth days 5 and 10 were observed at 885, 932, 1080, 1101, 1118, and 1150 cm^–1^. Together, these peaks represent C–H bending, ring breathing, and C–O–C vibrations of polysaccharides. The peaks at 1118 and 1150 cm^–1^ were assigned to cellulose and aliphatic C–O stretching in carbohydrates, respectively, and increased from day 5 to 10. For all strains, there was a significant increase in the 1158 cm^–1^ band, from day 5 to 10. This band corresponds to the asymmetric stretch of −C–O–C linkages, connecting monomers in polymeric compounds. In this case, this linkage was found within polysaccharides and the increase of this linkage could translate to a higher degree of polymerization of the polysaccharides, to more branching, and/or to a change in the type of polysaccharides within the cell wall (i.e., different polymers entirely with different chains).

The amide II band is considered to be one of the most reliable regions for the quantitative estimation of protein content.^32^ On day 5, the cell walls of *C. vulgaris* (13-1), *H. pluvialis*, and *Coelastrella* sp. (3-4) contained very similar quantities of protein, whereas *Scenedesmus* sp. (B2-2) contained less. On day 10, all strains contained comparable quantities of protein, meaning that the total protein content of *Scenedesmus* sp. (B2-2) cell wall significantly increased with time. For the other three strains, the protein to carbohydrate ratio significantly lowered from day 5 to 10, meaning that the cell wall changes in composition between growth phases.

### Outer Surface of Algal Strains Differs in Composition between Strains, But Not between Growth Phases within the Same Strain

X-ray photoelectron spectroscopy (XPS) is an advantageous technique for analyzing the biochemical composition of a cell surface as it is highly sensitive, nondestructive, and relatively quick. This technique can quantify lipids, proteins, and polysaccharides within the outer first 10 nm of the cell’s surface, without interference from the bulk of the microbial particle.^33^ Cryo-XPS, however, is less common and involves rapidly freezing the wet sample to liquid nitrogen temperatures, and maintaining the sample at extremely low temperatures throughout the duration of the experiment. In the last few years, cryo-XPS has commonly been used for the study of fungal or bacterial cell walls.^34^ In the present study, cryo-XPS was used since the low temperature during analysis minimizes cell wall alterations and surface contaminations.^35^

**Figure 3:**
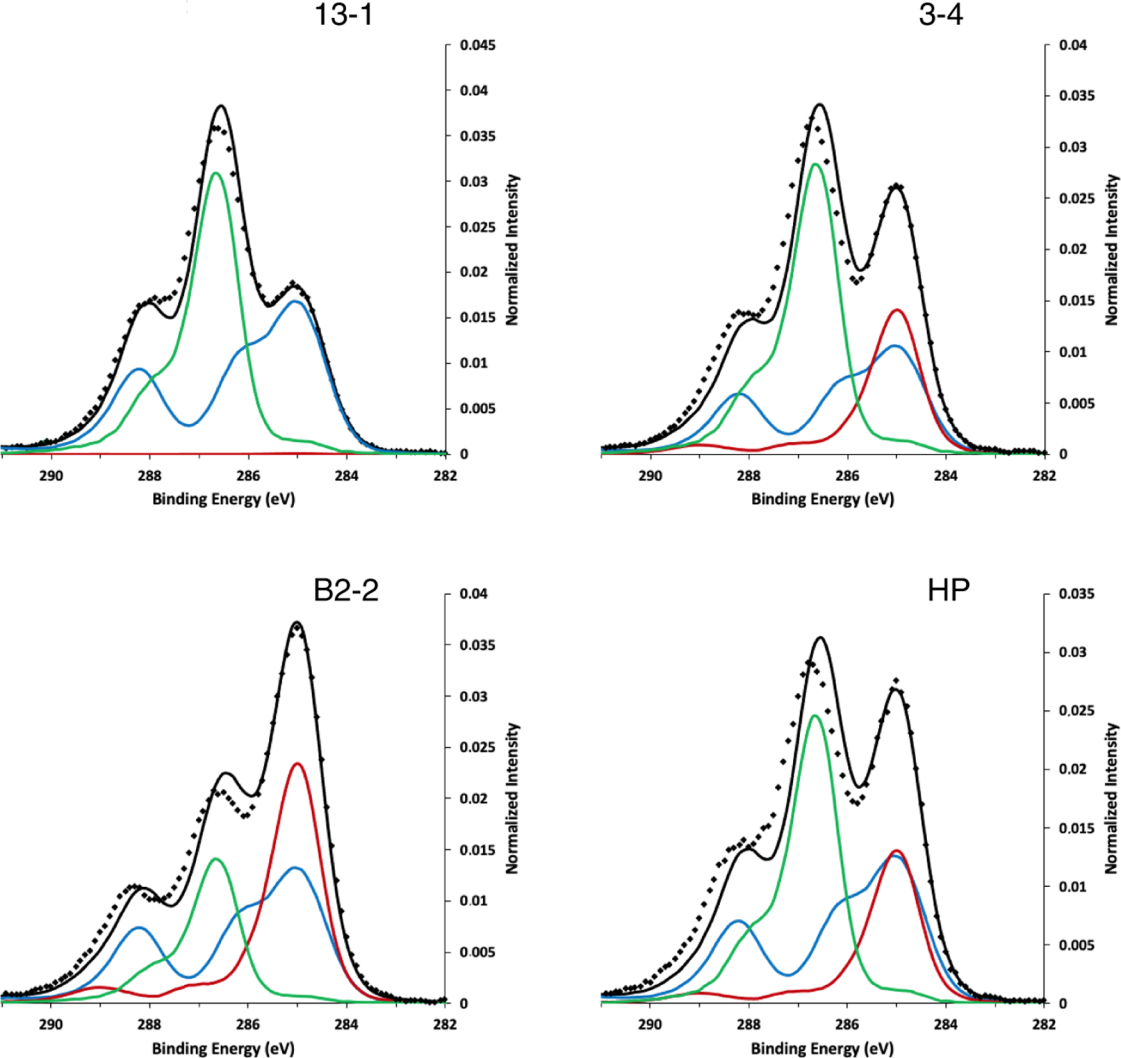
C1 spectra of *C. vulgaris* (13-1), *Coelastrella* sp. (3-4), *H. pluvialis* (HP), and *Scenedesmus* sp. (B2-2) in the logarithmic (day 5, dotted line) and stationary growth phase (day 10, solid line). The multivariate model has been performed on spectra of day 5; red: lipids, green: peptides, blue: carbohydrates.

C1 spectra were obtained from the microalgae grown in logarithmic and stationary growth phase (Figure 3). The C1 spectra obtained by cryo-XPS could be decomposed and assigned to three different compounds: lipids (red line), peptides (green line), and carbohydrates (blue line). Even though Ramstedt et al. originally developed the model to predict these three categories of compounds based on the multivariate analysis of the spectral components of Gram-negative bacteria, it was found to be sufficient to determine the microalgal cell wall composition.^14,36,37^

The four algal strains showed differences in composition between strains, but in three strains, no major differences were observed between growth phases within the same strain. The exception was for *C. vulgaris* (13-1), which showed an increase in lipids between day 5 and day 10 (Table 2). The outer cell wall of *C. vulgaris* (13-1) and *Coelastrella* sp. (3-4) displayed high quantities of polysaccharides, pointing to the hydrophilicity of the cell surface, which could provide colloidal stability of the algae in aqueous solutions.^14^ In comparison, *H. pluvialis* and *Scenedesmus* sp. (B2-2) had lower amounts of polysaccharides at their surface and higher quantities of proteins. The surface of *Scenedesmus* sp. (B2-2), in particular, contained significantly more proteins than any other compound and contained more lipids than carbohydrates. The teeth-like structures and spines on the outer surface of the cell wall of *Scenedesmus*, visible also in our TEM images of B2-2 (Figure 1U), are embedded into a pectic layer and have been reported to be composed of glycoproteins.^38^ This would explain the high quantity of proteins observed in the outer layer of the cell wall of the Nordic *Scenedesmus* strain B2-2.

**Table 2:** Relative Quantity of Polysaccharides, Lipids, and Proteins in the Outer 10 nm of the Cell Wall of *C. vulgaris* (13-1), *Coelastrella* sp. (3-4), *H. pluvialis* (HP), and *Scenedesmus* sp. (B2-2) Analyzed by Cryo-XPS

	13-1		3-4		HP		B2-2	
% of C atoms in	day 5	day 10	day 5	day 10	day 5	day 10	day 5	day 10
proteins	54	36	34	30	47	42	41	46
lipids	0	13	24	22	17	15	39	30
polysaccharides	46	51	42	48	36	43	20	24

### Cellulose Present in the Cell Wall of Logarithmic Growing *H. pluvialis* Changes into Mannose in the Stationary Phase

Our cryo-XPS measurements have revealed that the carbohydrate content in the cell wall of Nordic microalgae varies greatly between strains, but not so much between growth phases (Table 2). In the stationary growth phase, polysaccharides account for 51% of the carbon atoms in the cell wall in *C. vulgaris*, and 48, 43, and 24% for *Coelastrella* sp. (3-4), *H. pluvialis*, and *Scenedesmus* sp. (B2-2), respectively. A detailed analysis of the cell wall carbohydrates revealed that the cell walls of Nordic microalgae were composed of arabinose, rhamnose, fucose, xylose, mannose, galactose, galacturonic acid, glucose, and glucuronic acid (Table 3). The least abundant sugars were generally fucose, galacturonic acid, and xylose. The most abundant monosaccharides were, for all strains, rhamnose together with glucose. For two strains, in particular, *Coelastrella* sp. (3-4) and *Scenedesmus* sp. (B2-2), the glucose content varied to a high extent between growth phases. The quantity of glucose increased over time for *Coelastrella* sp. (3-4) from 8.99 ± 4.10 to 36.23 ± 8.50 μg/mg, whereas it significantly decreased for *Scenedesmus* sp. (B2-2) (from 66.33 ± 36.65 to 12.79 ± 3.17). This can be interpreted as an increase (or decrease, respectively) in cellulosic polymers in the cell wall. It is known that members of the *Scenedesmus* family, such as *Scenedesmus obliquus*, contain cellulose in the inner cell wall layers.^38^ However, cellulose has not been observed in the trilaminar outer layers of the cell wall that appear around the algal cells toward the stationary growth phase.^38^ The cellulose content over time in our Nordic strain therefore might decrease at the expense of other polysaccharides or glycoproteins that contribute to the formation of the rigid outer layers. Rhamnose has previously been reported to be the most abundant monosaccharide in the *C. vulgaris* cell wall, and rhamnose was reported to represent 33% of the total sugars in *Neochloris oleobundans* cell wall.^39^ The high quantity of rhamnose-containing polysaccharides may contribute to cell wall rigidity and resistance.^39^ Mannose was also present in high amounts in *Scenedesmus* sp. (B2-2) and *H. pluvialis*. Guo et al.^40^ recently discovered that the cellulose present in the flagellate cells of *H. pluvialis* gradually changed into mannose in the aplanospore stages. Our results support this discovery, as the glucose content in the *H. pluvialis* cell wall decreased from day 5 to 10, whereas the mannose content increased (Table 3). This fits well with the study conducted by Hagen and co-workers,^22^ which revealed high content of mannose (that could originate from mannan) in the cell wall, especially in the stationary growth phase. The formation of mannose could be a selfprotection mechanism that might help algal cells to survive and adapt to stressful environmental conditions. It could also be beneficial to the existence of astaxanthin.^40^

**Table 3:** Monosaccharide Analysis of the Cell Wall Determined by Trimethylsilyl (TMS) Derivatization and GC–MS

μg/mg DW cell wall		Ara	Rha	Fuc	Xyl	Man	Gal	GalA	Glc	GlcA
*C. vulgaris* (13-1)	day 5	2.87 ± 1.36	30.74 ± 3.26	0.80 ± 0.87	2.46 ± 0.75	0.95 ± 0.57	3.62 ± 0.08	2.91 ± 3.22	8.91 ± 1.86	3.77 ± 3.60
	day 10	4.29 ± 0.63	30.45 ± 3.65	0.37 + 0.20	1.63 ± 0.23	4.02 ± 0.35	10.01 ± 1.71	3.52 ± 0.53	9.26 ± 3.36	1.90 ± 0.82
*Coelastrella* sp. (3-4)	day 5	1.05 ± 0.15	25.31 ± 5.26	0.27 ± 0.11	0.97 ± 0.16	1.25 ± 1.20	2.66 ± 1.45	1.39 ± 0.50	8.99 ± 4.10	1.21 ± 0.92
	day 10	4.23 ± 1.18	31.18 ± 3.75	0.23 ± 0.11	1.65 ± 0.43	2.94 ± 0.54	10.64 ± 1.85	4.36 ± 0.31	36.23 ± 8.50	1.58 ± 0.50
*Scenedesmus* sp. (B2-2)	day 5	0.49 ± 0.25	15.44 ± 2.16	0.44 ± 0.05	0.44 ± 0.17	35.09 ± 19.54	0.30 ± 0.27	0.91 ± 0.23	66.33 ± 36.65	1.25 ± 0.33
	day 10	0.69 ± 0.39	16.31 ± 2.59	0.99 ± 0.28	0.67 ± 0.29	34.52 ± 4.57	0.79 ± 0.12	1.20 ± 0.49	12.79 ± 3.17	1.57 ± 0.79
*H. pluvialis* (HP)	day 5	1.59 ± 0.44	24.20 ± 4.23	0.31 ± 0.15	1.32 ± 0.33	17.60 ± 1.50	8.68 ± 0.76	3.03 ± 0.43	14.03 ± 0.69	1.18 ± 0.42
	day 10	0.75 ± 0.18	17.38 ± 4.78	0.34 ± 0.29	0.74 ± 0.04	21.35 ± 2.86	1.26 ± 1.72	1.31 ± 0.41	9.33 ± 2.17	1.38 ± 1.14

### *Scenedesmus* sp. (B2-2) Produces More Polar Amino Acids as It Evolves into the Stationary Phase

**Figure 4:**
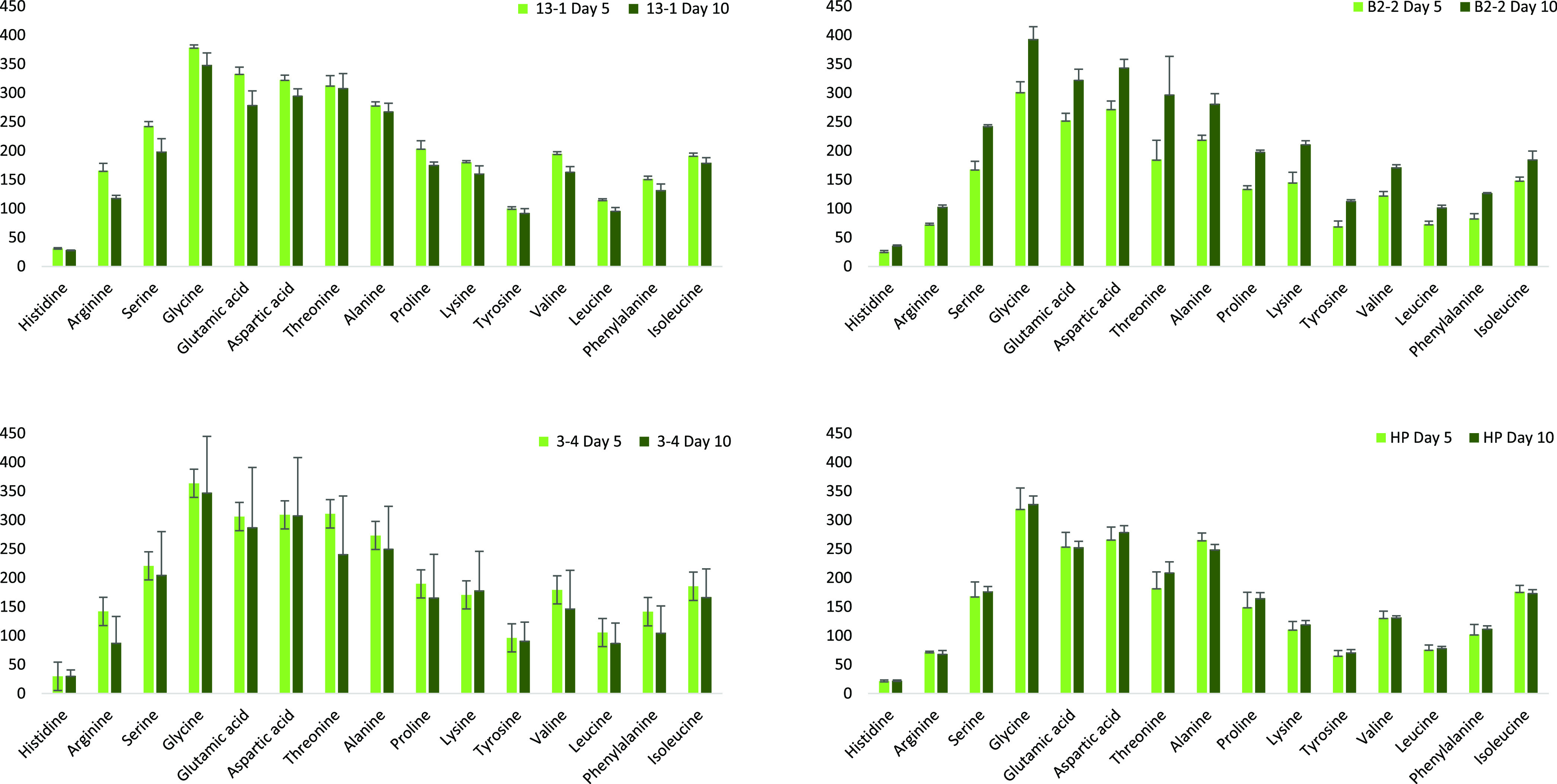
Amino acid profiles of the cell walls of *C. vulgaris* (13-1), *Coelastrella* sp. (3-4), *H. pluvialis* (HP), and *Scenedesmus* sp. (B2-2) on growth days 5 (light green) and 10 (dark green). The error bars represent the deviation between triplicates (*n* = 3).

We studied the amino acid profiles to get an understanding of the types of proteins that make up the cell wall. Amino acids can be hydrophobic or hydrophilic, and cell surface hydrophobicity is a factor that can strongly influence flocculation, a popular algae harvesting technique. Higher quantities in certain amino acids, such as leucine, for example, can indicate certain biological roles within the cell wall, as leucine-rich repeat proteins are known to play a vital role in stress responses. The amino acid profiles of the cell wall proteins of Nordic strains grown in the logarithmic and stationary phases are shown in Figure 4. *C. vulgaris* (13-1) and *Coelastrella* sp. (3-4) had comparable profiles, both in the exponential and stationary growth phases. Their total amino acid content in the cell wall decreased as the cultures got older, which confirms our FTIR data, in which the protein:carbohydrate ratio decreased over time. Glycine, glutamic acid, aspartic acid, threonine, and alanine are the most abundant amino acids in the cell walls of all four strains. There seems to be no clear tendency for the cell walls to contain more polar or nonpolar amino acids; however, *Scenedesmus* sp. (B2-2) produced more polar than nonpolar amino acids as the culture entered the stationary phase (Table 4). For B2-2 and HP, the quantity of amino acids increased with time. This is true for B2-2 in particular, where the quantity of glycine, aspartic acid, and glutamic acid increased by 30, 26, and 27%, respectively, from growth day 5 to growth day 10. Cell walls of *Scenedesmus* sp. and *C. reinhardtii* have previously been reported to show structural similarities.^38^ The cell wall of *C. reinhardtii* contains glycoproteins that, in turn, contain high proportions of glycine.^38^ If we assume that the glycine in B2-2 cell walls also originates from glycoproteins, we can conclude that their quantity in the cell wall is higher in the stationary phase than in the exponential phase.

**Table 4:** Amino Acid Content of the Cell Wall Based on Polarity and Side-Chain Charge

		polar (nmol/mg)	nonpolar (nmol/mg)	positive side chain (nmol/mg)	negative side chain (nmol/mg)	neutral (nmol/mg)	sum (nmol/mg)
13-1	day 5	1688.3	1512.7	376.9	655.7	2168.4	6401.9
	day 10	1481.5	1363.6	307.4	574.6	1963.2	5690.2
B2-2	day 5	976.85	1081.6	242.7	524.7	1503.5	4329.5
	day 10	1670.3	1458.4	350.2	666.8	2111.6	6257.4
3-4	day 5	1526.7	1408.4	318.9	620.2	1996.0	5870.2
	day 10	1353.3	1369.9	285.9	564.8	1872.6	5446.4
HP	day 5	1138.7	1217.8	203.0	520.4	1633.1	4713.0
	day 10	1199.3	1237.1	210.0	532.2	1694.2	4872.8

In conclusion, this publication is the first to give in-depth insight into the cell wall composition of microalgal strains used in biotechnological applications.

Through various characterization methods, including FTIR spectroscopy and Cryo-XPS, and in combination with transmission electron microscopy, we were able to report the composition and morphology of the cell walls of the Nordic strains *C. vulgaris* (13-1), *Scenedesmus* sp. (B2-2), *H. pluvialis* (HP), and *Coelastrella* sp. (3-4). The use of these different methodologies provides an overall view on the composition and structure of the cell walls. Cryo-XPS provides qualitative information on the protein, carbohydrate, and lipid content within the outermost layers of the cell wall (i.e the surface), which gives insight into how the algal cells can communicate with their surrounding environment. FTIR spectroscopy gives similar information, but this time on whole cell wall material. The results from Cryo-XPS and FTIR spectroscopy show the cell walls contain very low quantities of lipids but high quantities of carbohydrates and proteins. Therefore, we made a more in-depth analysis of these two components, by analyzing the monosaccharide and amino acid profiles of the cell walls. TEM allowed us to image visual changes in the cell wall between growth phases, as well as to measure the thickness of the cell walls. The results are summarized in Table 5.

**Table 5:**
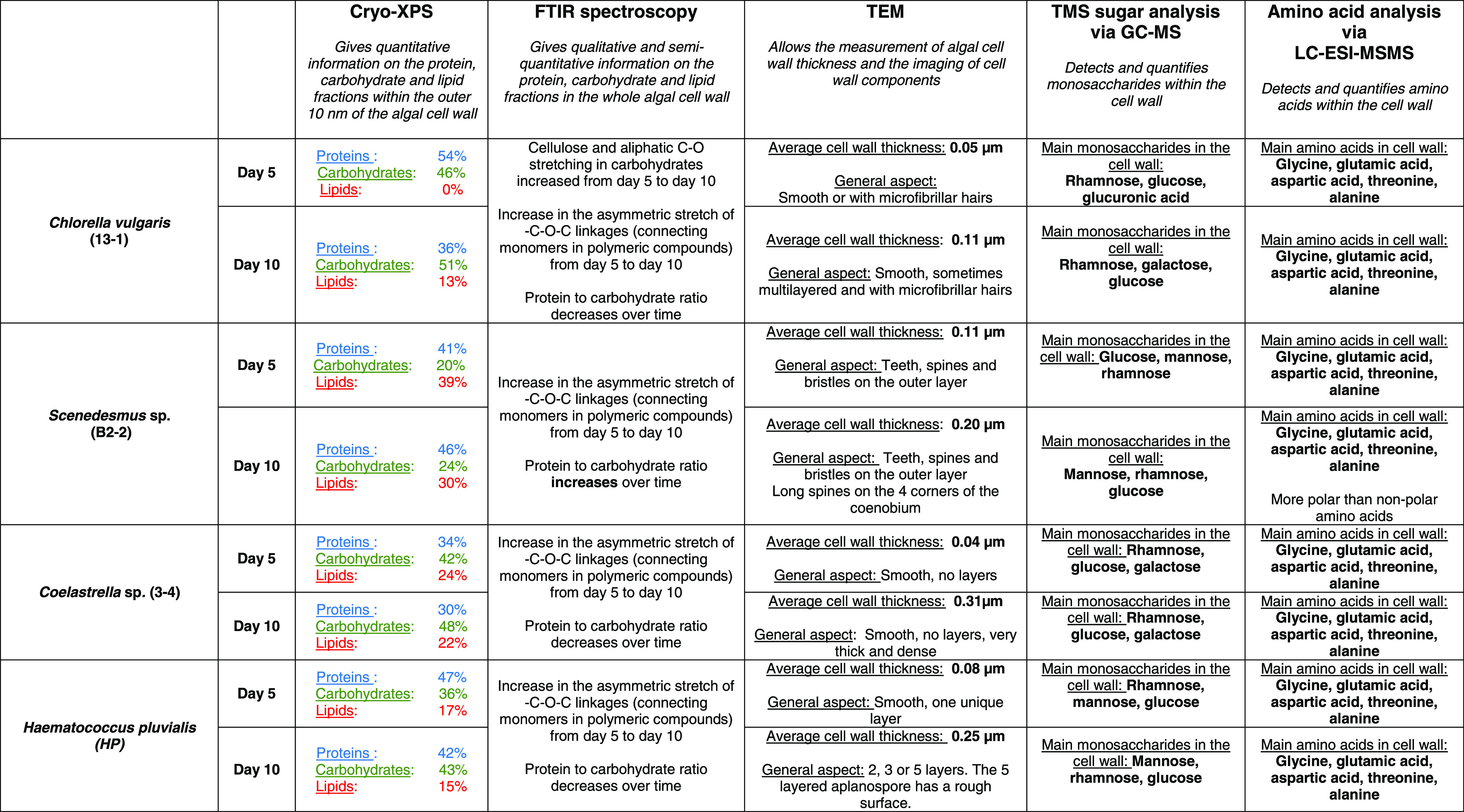
Summary of Cell Wall Composition and Characteristics of *C. vulgaris* (13-1), *Scenedesmus* sp. (B2-2), *Coelastrella* sp. (3-4), and *H. pluvialis* (HP)

FTIR spectroscopy showed that the protein/carbohydrate ratio considerably varies with the growth phase. For *C. vulgaris* (13-1), *H. pluvialis* (HP), and *Coelastrella* sp. (3-4), the total protein content in the cell wall decreased with time, whereas for *Scenedesmus* sp. (B2-2), it increased. The structures and spines that appear in the stationary growth phase on the surface of *Scenedesmus* sp. (B2-2) cells after the formation of the coenobium seem to contain high quantities of protein. Cryo-XPS gave an understanding of the surface composition of the four strains. It confirmed the unique characteristics of the cell wall surface of *Scenedesmus* sp. (B2-2), which has a high lipid and protein content in comparison with the other strains. GC analysis of the cell wall monosaccharides revealed high quantities of rhamnose, mannose, and/or glucose, depending on the strain.

The results presented here will contribute to the choice and optimization of downstream processing technologies. Using chemical cell wall disruption, the selectivity, suitability, and efficiency of, for example, surfactants, solvents, or antibiotics, are largely dependent on the microalgal cell wall composition and structure.^41^ During biological disruption, such as enzymatic lysis, knowledge of cell wall composition will enable us to select enzymes to target specific compounds within the cell wall.

The data also are of relevance for the choice of the harvesting technology, e.g., the cell wall carbohydrate composition helps to understand flocculation. The total carbohydrate content was found to be the most significant factor positively affecting chitosan flocculation.^42^ Our results therefore give a good understanding of the cell wall properties of Nordic strains and can be used not only to solve the current challenges in current biotechnological processes but also to get a better understanding of the evolution and diversity of green microalgae.

## Supporting Information Available

Growth curves of *C. vulgaris* (13-1) (A), *Scenedesmus* sp. (B), *H. pluvalis* (C), and *Coelastrella* sp. (3-4) (D) (Figure S1) (PDF)
