# Supplementary material for: Detailed Characterization of the Cell Wall Structure and Composition of Nordic Green Microalgae
Source: J Agric Food Chem. 2022 Jul 27;70(31):9711–21. doi: 10.1021/acs.jafc.2c02783 (PMC9372998; doi:10.1021/acs.jafc.2c02783)
Supplement: Supplementary file 1 — jf2c02783_si_001.pdf [file jf2c02783_si_001.pdf]

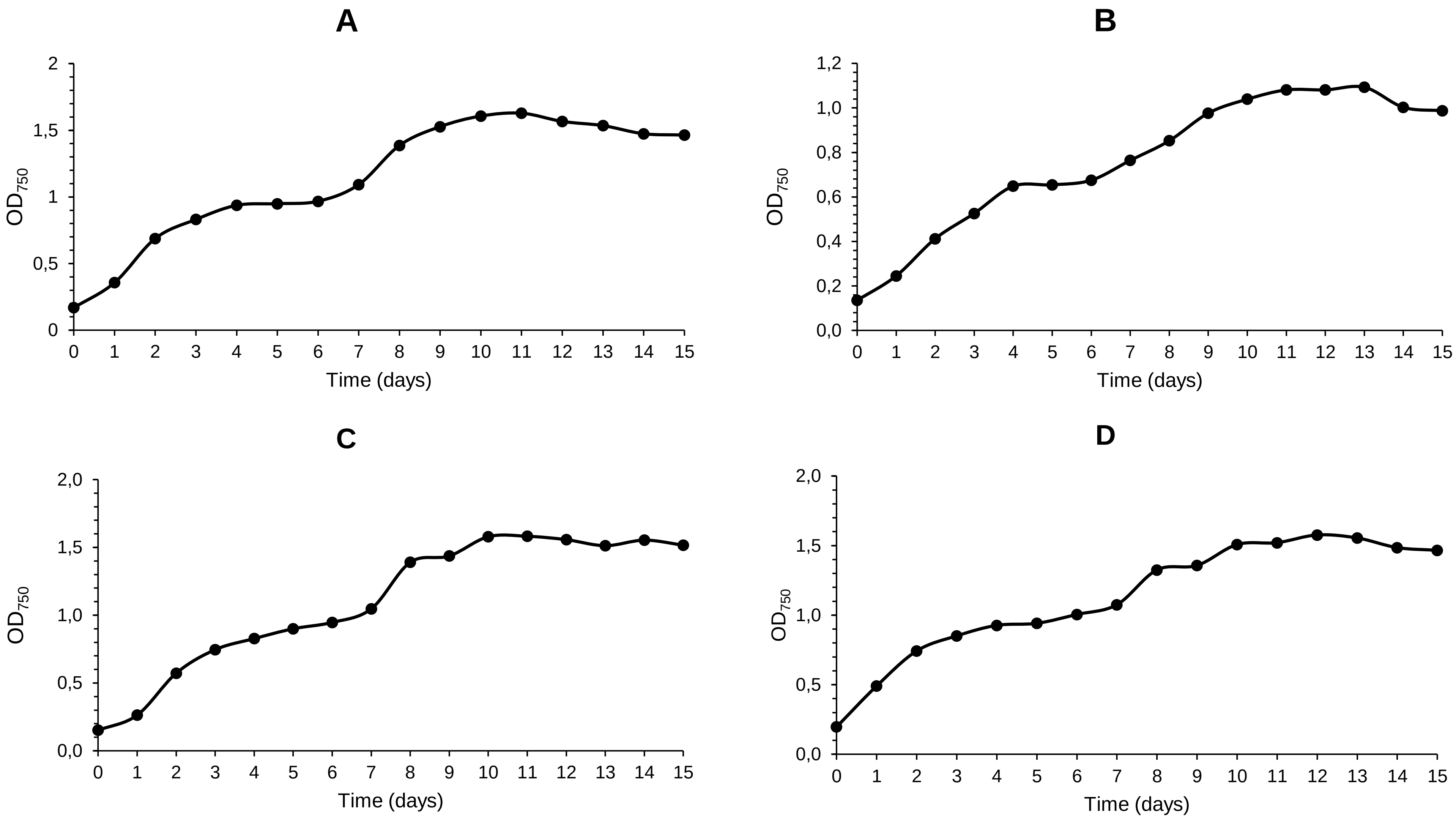

Supplementary Figure 1: Growth curves of *Chlorella vulgaris (13-1)* (***A***), *Scenedesmus sp.* (***B***), *Haematococcus pluvalis* (***C***) and *Coelastrella sp. (3-4)* (***D***)
